# The Green Synthesis of Reduced Graphene Oxide Using Ellagic Acid: Improving the Contrast-Enhancing Effect of Microbubbles in Ultrasound

**DOI:** 10.3390/molecules28227646

**Published:** 2023-11-17

**Authors:** Qiwei Cheng, Yuzhou Wang, Qi Zhou, Shaobo Duan, Beibei Zhang, Yaqiong Li, Lianzhong Zhang

**Affiliations:** 1 Zhengzhou University People’s Hospital, Henan Provincial People’s Hospital, Zhengzhou University, Zhengzhou 450052, China; chengqwe@gs.zzu.edu.cn (Q.C.); wangyuzhou1221@haue.edu.cn (Y.W.); zq0830@gs.zzu.edu.cn (Q.Z.); 2 Department of Ultrasound, Henan Provincial People’s Hospital, Zhengzhou 450003, China; dustin2662@zzu.edu.cn (S.D.); liyaqiong@zzu.edu.cn (Y.L.); 3 Henan Engineering Technology Research Centre of Ultrasonic Molecular Imaging and Nanotechnology, Henan Provincial People’s Hospital, Zhengzhou 450003, China; zhangbb@haut.edu.cn

**Keywords:** reduced graphene oxide, ellagic acid, mechanical index, ultrasound contrast agent, microbubble

## Abstract

There is an urgent need to realize precise clinical ultrasound with ultrasound contrast agents that provide high echo intensity and mechanical index tolerance. Graphene derivatives possess exceptional characteristics, exhibiting great potential in fabricating ideal ultrasound contrast agents. Herein, we reported a facile and green approach to synthesizing reduced graphene oxide with ellagic acid (rGO-EA). To investigate the application of a graphene derivative in ultrasound contrast agents, rGO-EA was dispersed in saline solution and mixed with SonoVue (SV) to fabricate SV@rGO-EA microbubbles. To determine the properties of the product, analyses were performed, including ultraviolet–visible spectroscopy (UV–vis), Fourier-transform infrared spectroscopy (FTIR), Raman spectroscopy, transmission electron microscopy (TEM), thermal gravimetric analysis (TGA), X-ray photoelectron spectrum (XPS), X-ray diffraction analysis (XRD) and zeta potential analysis. Additionally, cell viability measurements and a hemolysis assay were conducted for a biosafety evaluation. SV@rGO-EA microbubbles were scanned at various mechanical index values to obtain the B-mode and contrast-enhanced ultrasound (CEUS) mode images in vitro. SV@rGO-EA microbubbles were administered to SD rats, and their livers and kidneys were imaged in CEUS and B-mode. The absorption of rGO-EA resulted in an enhanced echo intensity and mechanical index tolerance of SV@rGO-EA, surpassing the performance of SV microbubbles both in vitro and in vivo. This work exhibited the application potential of graphene derivatives in the field of ultrasound precision medicine.

## 1. Introduction

The past decades have witnessed enormous advancements in graphene research since the initial isolation of this two-dimensional miracle material. As an allotrope of carbon containing sp2-bonded atoms, graphene has impressive features, such as exceptional mechanical, electrical, optical and thermal characteristics. The ever-increasing interest in graphene-based materials has been focused on biomedical science in recent years. Additionally, inspirational research on graphene derivatives has been focused on wearable electronics, diagnostic tools, drug-delivery carriers and regenerative medicine materials [1,2,3,4]. However, ultrasound medical science gains little attention in graphene research.

In the ultrasound medical area, contrast-enhanced ultrasound (CEUS) is an important imaging technique, specialized for acquiring sensitive blood flow and tissue perfusion information. Ultrasound contrast agents (UCAs), the coated microbubbles that are stable in vivo, can be used to induce cavitation for diagnostic and therapeutic applications with suitably moderate ultrasound energy. This contributes to the development of accurate and subtle CEUS imaging. For decades, further contributions to enhanced cavitation effects and therapeutic delivery have witnessed the significance of UCA in molecular imaging and precise treatment [5,6]. The application of UCAs epitomizes real-time, highly safe and clearly visible merits in CEUS [7].

SonoVue (SV) is a UCA approved by the Food and Drug Administration for liver ultrasound (US) imaging [8]. SV microbubbles (MBs) with a lipid shell and a gas core provide the accurate dynamic detection of microcirculation, demonstrating the ability to transform a thermal process into a safer mechanical process by using microbubbles as actuators of acoustic forces [9]. Wang et al. fabricated cellulose nanocrystalline-modified SonoVue MBs, which improved the ultrasound imaging quality with a biologically renewable nanomaterial [10]. At a low mechanical index, steady-state cavitation mostly occurs in MBs, resulting in lasting enhancement. When a higher mechanical index is applied, the ultrasonic energy is sufficient to rupture smaller-sized MBs, leading to shorter acoustic enhancement [11]. Thus, there is still an urgent need for ideal UCAs that provide higher echo intensity and mechanical index (MI) tolerance. 

Green reducing agents, such as edible sunflower oil, lemon grass extracts and green tea extracts, can be employed for the eco-friendly synthesis of rGO [12,13,14]. Ellagic acid (EA), a non-toxic dietary polyphenol present in various fruits and nuts, possesses inherent abilities including antibacterial, anticancer, antioxidant and anti-inflammatory properties [15,16,17,18]. In addition, EA may potentially serve as a green reducing agent. Phenolic hydroxyl groups in EA will be transformed to corresponding quinone forms during the reduction [19]. Meanwhile, non-covalent bond functionalization between rGO and EA is achieved through interactions such as hydrogen bonds and π–π bonds. The green reducing compound works to stabilize and cap agents for the formation and stabilization of rGO [20].

In this work, we have explored the utilization of ellagic acid to develop a one-step, facile and eco-friendly approach for the preparation of rGO, avoiding expensive equipment, the need for a rigorous environment and the presence of toxic constituents inherent in conventional chemical synthesis [21]. Carbon nanomaterials are attracting great attraction in extensive fields [22]. With conditions of a rich stock in nature and considering the excellent inherent abilities of EA, the potential of the nano-scaled rGO-EA is promising. Moreover, the biosafety of the product has been evaluated through the cell viability assay and hemolysis assay. To our knowledge, few studies have explored the application of graphene in enhancing ultrasound diagnosis. This study aims to bridge the literature gap summarized above. We researched the combination of green-synthesized rGO-EA and SV for better contrast-enhancing effects and MI tolerance, and CEUS imaging quality was evaluated by acquiring and analyzing US imaging. Furthermore, rGO-EA-modified microbubbles can provide fundamental knowledge for the applications of graphene derivatives in ultrasonic diagnostic systems.

## 2. Results and Discussion

### 2.1. Characterization of rGO-EA

As a natural polyphenol, ellagic acid exhibits reduction properties. The GO solution with EA underwent sonication and exhibited an initial brown coloration. Upon completion of the reaction, the solution turned a deep, dark hue. 

**Figure 1:**
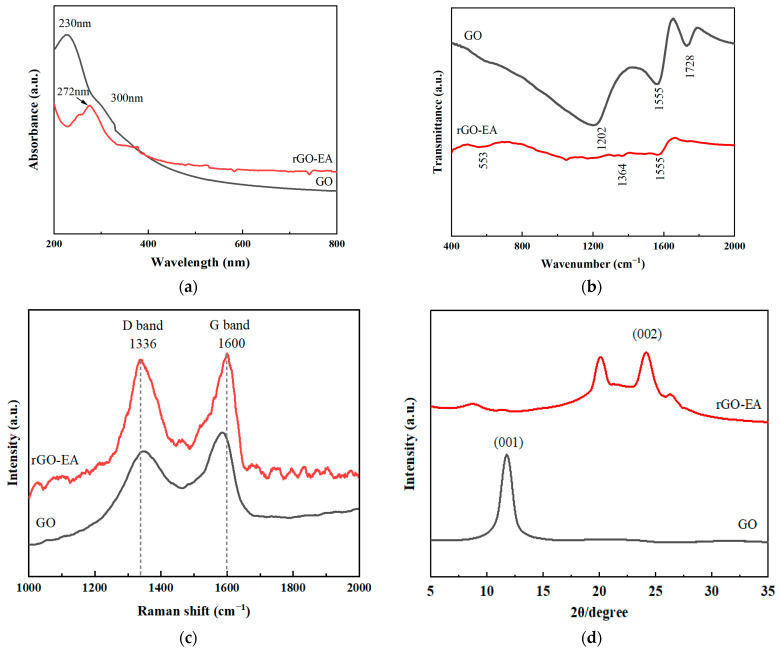
The UV–vis (**a**), FTIR (**b**), Raman (**c**) and XRD (**d**) spectra of GO and rGO-EA.

A series of characterizations was conducted to confirm the properties of the product. The reduction was monitored by measuring the position of the UV–vis absorption peak (Figure 1a). The UV–vis spectrum of GO exhibited a characteristic peak at 230 nm, corresponding to a π → π* transition of aromatic C=C bonds in the aromatic ring [23]. Additionally, a shoulder peak at ~300 nm was observed, which could be attributed to n → π* transitions of C=O and C–O groups present on graphene sheets [24]. As for rGO-EA, the absorption peak of primordial GO at 230 nm was eliminated, and the peak of graphene carbon skeleton red shifted to 276 nm, indicating the restoration of electronic bonding within the graphene sheets [25]. The reduction and the functionalization of rGO-EA were also investigated using FTIR spectroscopy (Figure 1b). The FTIR spectrum of GO exhibited a characteristic peak, 1728 cm^−1^, corresponding to the C=O stretching of carboxylic and/or carbonyl moiety functional groups, along with a peak at 1202 cm^−1^ associated with C–O stretching vibrations. Additionally, the presence of unoxidized graphitic domains was evidenced by the peak observed at 1555 cm^−1^ due to the skeletal vibrations [25,26]. The FTIR curve confirmed the presence of oxygen-containing functional groups in GO. After the reduction of GO using ellagic acid, distinct IR peaks of rGO-EA were observed at 1364 and 553 cm^−1^, which were associated with the characteristic peaks of EA, suggesting that EA was successfully conjugated [26]. To confirm the structural changes, Raman spectroscopy was employed with the as-received GO and the composite rGO-EA. The Raman spectra of GO and rGO-EA in the spectral region of 1000–2000 cm^−1^ are presented in Figure 1c. Two fundamental vibrations were observed for the G band at 1584 cm^−1^ and the D band at 1348 cm^−1^, indicating the presence of amorphous carbon [22]. The D band corresponded to the A_1g_ mode of k-point photons, and the G band in E_2g_ mode was associated with the ordered sp2-bonded carbon atoms in rGO [27]. The intensity of the G band was found to be higher than that of the D band, and the ID/IG value for GO was determined to be 0.83. In contrast, rGO-EA exhibited distinct bands at 1336 and 1600 cm^−1^, corresponding to the D and G bands. The calculated ID/IG value for rGO-EA was 0.96, surpassing that of GO, indicating the functionalization of rGO [26]. The crystalline nature of GO and rGO-EA was confirmed using XRD, and their spectra are shown in Figure 1d. GO exhibited a characteristic carbon peak (001) at ~11.78° [28]. As GO underwent reduction with EA, the peak at around 11.78° decreased, indicating the successful removal of most oxygen functional groups and the effective reduction of GO [29]. A new peak emerged at approximately 20.09°. The typical diffraction peak of rGO nanosheets was observed at around 26.57° for rGO-EA [30]. This result confirmed the restoration of the original graphite structure through reduction [29]. In addition, rGO-EA showed a distinct peak (002) at ~24.14°, which was in excellent agreement with the reported values for rGO [31].

**Figure 2:**
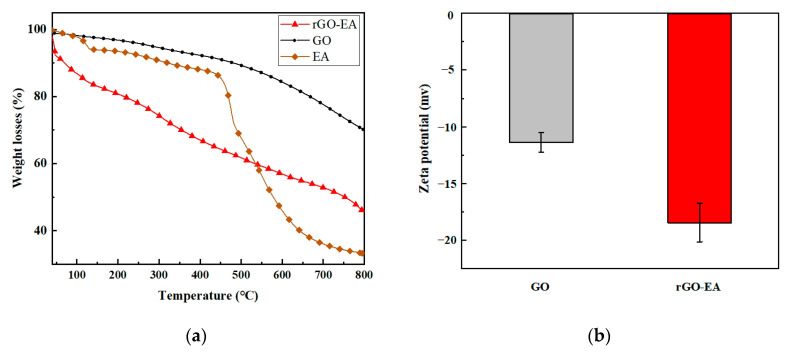
(**a**)The TGA curves of GO, EA and rGO-EA. The mass losses were observed between 40 °C and 800 °C. (**b**) Zeta potential of GO and rGO-EA was determined at 25 °C (*n* = 5).

The TGA method demonstrated high precision in examining the composition and thermal stability changes in composites. The TGA thermograms illustrating the characteristics of GO, EA and rGO-EA are presented in Figure 2a. The percentage of mass loss below 100 °C could be attributed to H2O evaporation, and the TGA measurement was employed to analyze the mass of adsorbed EA. Since EA was adsorbed due to the π-π interaction between EA and rGO, the significant mass loss percentage shown in the TGA thermogram between 40 °C and 800 °C corresponded to the decomposition of EA. The mass losses for the GO were less than that for the rGO-EA, confirming that the EA was loaded onto the surface of GO. Zeta potential values of the aqueous dispersions of GO and rGO-EA are shown in Figure 2b. It was found that the zeta potential of the GO and rGO-EA was negative. For GO, the initial zeta potential was approximately −11.3 mV. The zeta potential value of rGO-EA ascended to about −18.4 mV, indicating that the reduction process removed the oxygen-containing groups from the rGO-EA [32]. 

**Figure 3:**
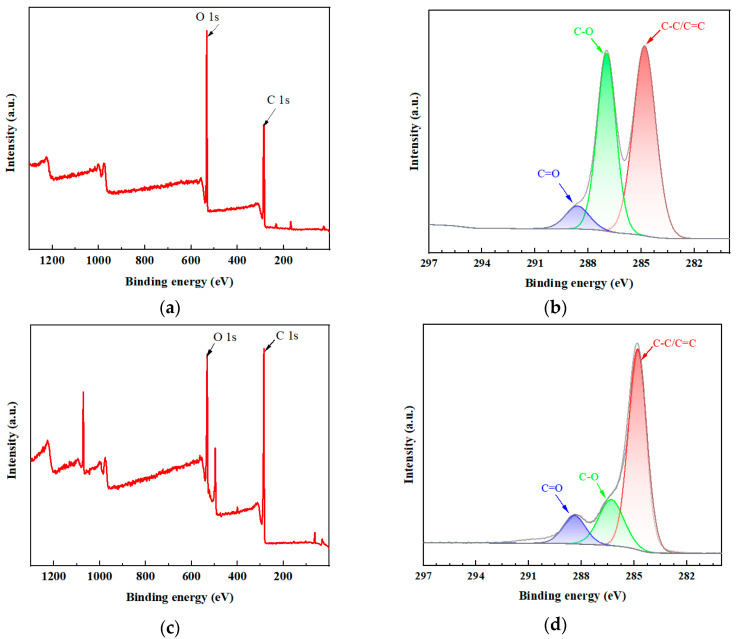
The XPS survey spectra of GO (**a**) and rGO-EA (**c**). The deconvoluted C 1s spectra of GO (**b**) and rGO-EA (**d**).

An XPS analysis was carried out to analyze the impact on the carbon and oxygen contents of the reduction process. Figure 3 shows XPS full spectra and the deconvoluted C 1s spectra of GO and rGO-EA. The deconvoluted C 1s peaked after Gaussian–Lorentzian fitting using peak processing software. The C1s peaks of GO (Figure 3a) with binding energies of ~284.8, ~287 and ~288.8 eV were associated with the carbon atoms in aromatic rings (C–C/C=C), C–O and C=O, respectively [33]. By contrast, the peak ratio of C–C/C=C content increased in rGO-EA, and the ratios of the oxygen functional group contents decreased (Figure 3c). This was consistent with the XRD results (Figure 1d). The specialized characterization of GO and rGO-EA is demonstrated in Figure 1, Figure 2 and Figure 3, showcasing the successful preparation and functionalization of rGO-EA.

### 2.2. Morphology of GO and rGO-EA

**Figure 4:**
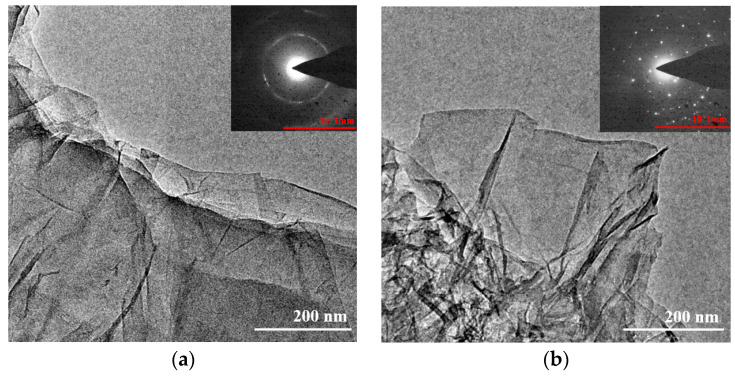
TEM images of GO (**a**) and rGO-EA (**b**) with the SAED pattern inset.

TEM examinations were carried out to describe the morphological characteristics of GO and rGO-EA. The SAED patterns of GO and rGO-EA are also shown in Figure 4. The TEM image revealed the nanosheet of GO, along with paper-like and transparent features (Figure 4a). In addition, the two-dimensional single-layer synthesized rGO-EA (Figure 4b) with nanoscale morphology was clearly visible. After the reduction, the rGO-EA exhibited scrolled and wrinkled features, which also certified the successful functionalization. The SAED pattern of rGO-EA reveals the signature of EA onto the surface of rGO [34].

### 2.3. Cell Viability Assay

**Figure 5:**
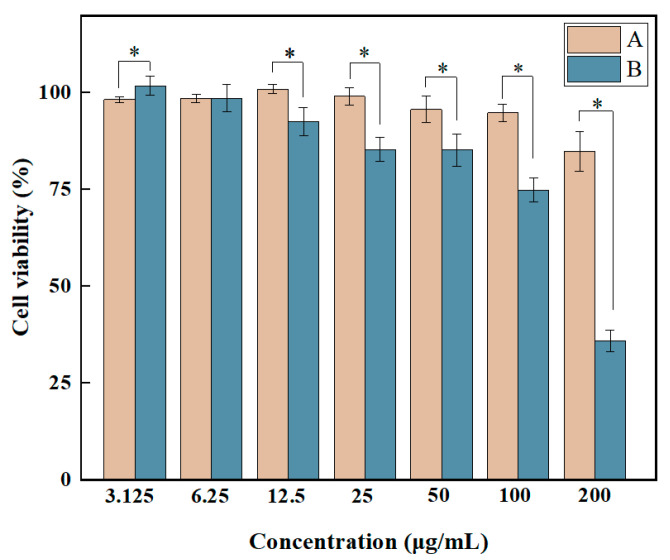
The cell viability of NIH3T3 and 4T1 cells treated with rGO-EA. Group A: NIH3T3 cells. Group B: 4T1 cells. The concentration of rGO-EA varied from 3.125 to 200 μg/mL. Cells incubated in complete medium were set as a control. *: *p* < 0.05 between NIH3T3 and 4T1 groups (*n* = 6).

The in vitro cell viability of rGO-EA on the 4T1 breast cancer cell line and the NIH3T3 fibroblast cell line were obtained using CCK8 analysis to evaluate the metabolic activity of cultured cells. IBM SPSS Statistics 22 software was utilized to determine the half-maximal inhibitory concentration (IC50) of rGO-EA against 4T1 cells. As shown in Figure 5, a clear dose-dependent growth inhibition was observed for the green-synthesized rGO-EA on 4T1 cells. The viability of these cells decreased with the increasing concentration of rGO-EA. For 4T1 cells, the IC50 of rGO-EA was 172.43 ± 20.62 μg/mL. At the maximum concentration (200 μg/mL) of rGO-EA, approximately 35.9% cell inhibition was observed, indicating that rGO-EA possessed growth-inhibiting ability and therapeutic potential on 4T1. At a concentration < 200 μg/mL, rGO-EA showed little influence on NIH3T3, and 4T1 exhibited higher sensitivity. 

### 2.4. Hemolysis Assay

**Figure 6:**
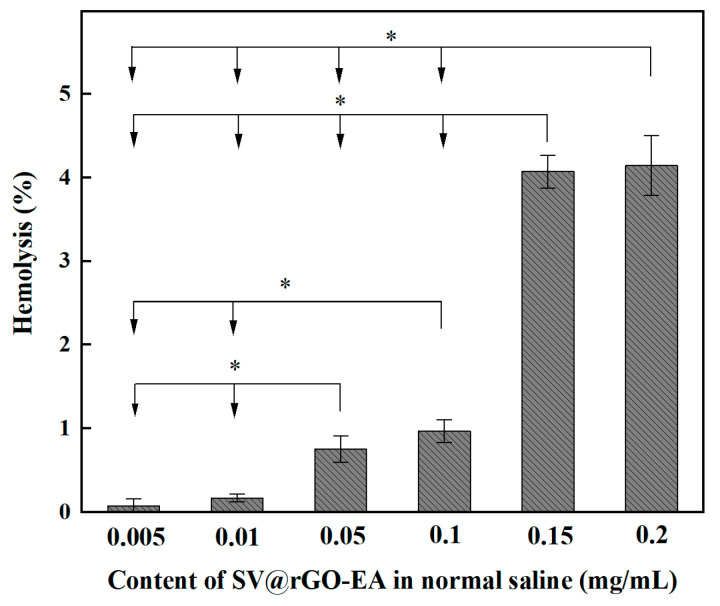
The hemolysis of RBCs incubated with rGO-EA for 4h at 37 °C. *: *p* < 0.05 compared with other groups, in which the arrow pointed to (*n* = 5).

Hemolysis is attributed to the damage of the RBC membrane, leading to hemoglobin being released into the surrounding solution. Generally, a hemolysis level less than 5% indicates a feasible biomaterial [35]. Figure 6 shows the hemolysis of the RBCs incubated with varying concentrations of rGO-EA for 4h at 37 °C. The hemolysis (%) of the rGO-EA suspension was calculated according to the equation *Hemolysis* (%) = (*ODe* − *ODn*)/(*ODp* − *ODn*) × 100%, where *ODe*, *ODn* and *ODp* correspond to the absorbance values of the experimental, negative and positive groups, respectively. Overall, hemolysis increased with the rising SV@rGO-EA concentration (up to 4.1% at 0.2 mg/mL), but this was insignificant (<5%).

### 2.5. Morphology and Zeta Potential Values of MBs

**Figure 7:**
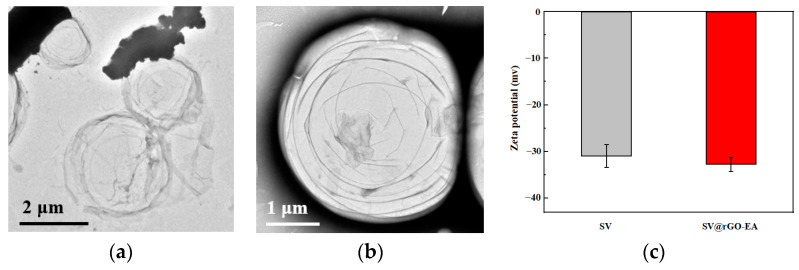
(**a**) TEM images of SV. SV MBs displayed spherical surfaces. (**b**) TEM images of SV@rGO-EA. rGO-EA could be clearly seen on the surface of SV. (**c**) Zeta potential values of SV and SV@rGO-EA were measured at 25 °C (*n* = 5).

The TEM image reveals the spherical morphology of the SV MBs in Figure 7a. To demonstrate adherence, the TEM image of SV@rGO-EA in Figure 7b exhibits single-layer graphene sheets on the smooth surface of MBs. The presence of rGO-EA could be identified by the delicate lateral edges. The zeta potential distribution of the aqueous dispersions of SV and SV@rGO-EA MBs are shown in Figure 7c. At pH = 7, the net charge of SV was −30.9 mV, whereas the zeta potential value of SV@rGO-EA was −32.7 mV, confirming the stable combination of rGO-EA and the excellent suspension stability of SV@rGO-EA MBs at pH = 7 [36,37].

### 2.6. In Vitro Ultrasound Imaging

**Figure 8:**
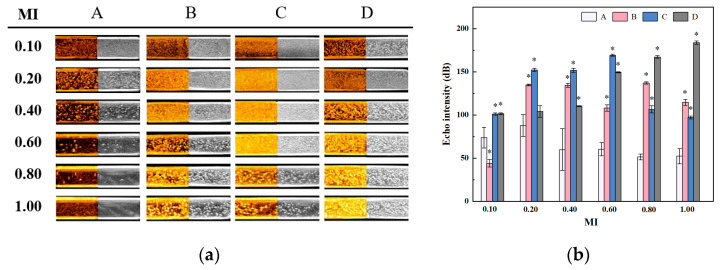
(**a**) The CEUS and B-mode ultrasound images of contrast agents in vitro. (**b**) Echo intensities of groups in conditions of different MIs. Group A: control, which contained SV only. Groups B–D: SV@rGO-EA groups, where the concentrations of rGO-EA were 0.05, 0.10 and 0.20 mg/mL, respectively. *: *p* < 0.05 compared with the SV group (*n* = 3).

The CEUS and B-mode ultrasound images of SV and SV@rGO-EA are presented in Figure 8a. Pipettes comprising SV in group A were set as a control. The acoustic signal of SV peaked at a MI of 0.20, and it gradually faded away as the MI increased from 0.20 to 1.00, indicating that SV exhibited its highest contrast-enhanced ability at a MI of 0.20. By contrast, SV@rGO-EA groups showed better MI tolerance and provided a better view than the SV group, which was obvious when the MI was over 0.40. The echo intensities of each group under different MIs are shown in Figure 8b. Consistent with Figure 8a, the echo intensity of the control diminished with the rise in MI from 0.20 to 1.00, whereas SV@rGO-EA groups exhibited consistently higher echo intensities overall. Notably, at rGO-EA concentrations of 0.10 and 0.20 mg/mL, better enhancement was observed compared to SV on CEUS images acquired with different MI values (0.10, 0.20, 0.40, 0.60, 0.80 and 1.00). This could be attributed to the exceptional mechanical characteristic of rGO-EA nanosheets adhering to the MBs, which might provide protection against the stronger ultrasonic energy at higher MI values.

### 2.7. In Vivo Ultrasound Imaging

**Figure 9:**
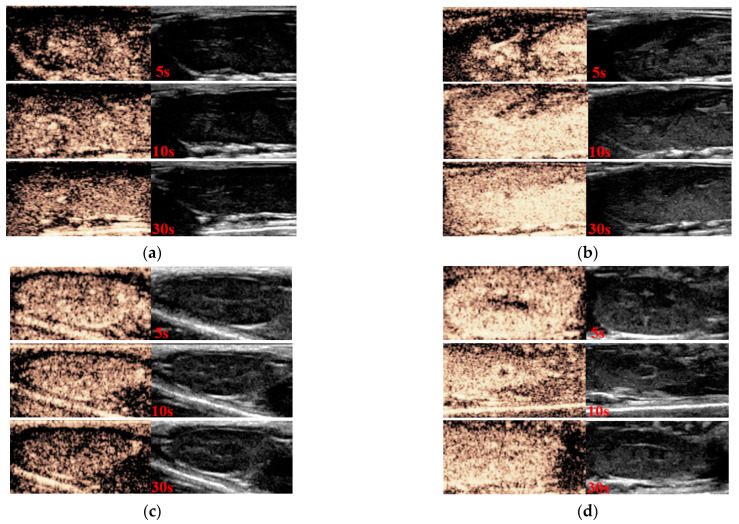
CEUS and B-mode US images of SV MBs in the liver (**a**) and kidney (**c**). CEUS and B-mode US images of SV@rGO-EA MBs in the liver (**b**) and kidney (**d**). Images were acquired after the intravenous injection at 5 s, 10 s and 30 s, respectively. The concentration of rGO-EA in SV@rGO-EA MBs was 0.10 mg/mL. The equivalent SV concentration was 5 mg/mL. The MI value was 0.08.

The ultrasound enhancement assessment of SV and SV@rGO-EA at 0.08 MI in the animal body was carried out using SV as the control group, and B-mode imaging was recorded at the same time in comparison with CEUS. We took the results of the biosafety assay and ultrasound imaging in vitro into consideration, and the rGO-EA concentration in SV@rGO-EA MBs was set at 0.10 mg/mL, which provided impressive echo intensity and CEUS imaging in vitro. The equivalent SV concentration was 5 mg/mL in every group. The CEUS and B-mode imaging effect on rat livers and kidneys is presented in Figure 9, following intraperitoneal injection at 5 s, 10 s and 30 s. The bright imaging effect on rat liver after the intraperitoneal injection of SV in CEUS is demonstrated in Figure 9a, and it exhibited a significant enhancement in liver parenchyma. In comparison, Figure 9b illustrates that SV@rGO-EA MBs provided a much superior enhancement effect in CEUS under ultrasonic irradiation within the liver parenchyma, which remained exceptionally pronounced even at 30 s. As for the kidneys, Figure 9c,d exhibits distinct boundaries of the kidneys in both CEUS and B-mode, and an evident enhancement in CEUS mode was observed. By contrast, SV@rGO-EA MBs demonstrated superior performance in renal CEUS imaging, according to Figure 9d. The renal echo exhibited a more pronounced contrast-enhancing effect than hepatic echo following early injection at 5 s, as depicted in Figure 9b,d. However, it was also evident that the enhancing signal of the kidneys dissipated at a faster rate compared to the liver. In summary, better hepatic and renal signals in CEUS were observed after injecting SV@rGO-EA, compared to the SV injection state.

**Figure 10:**
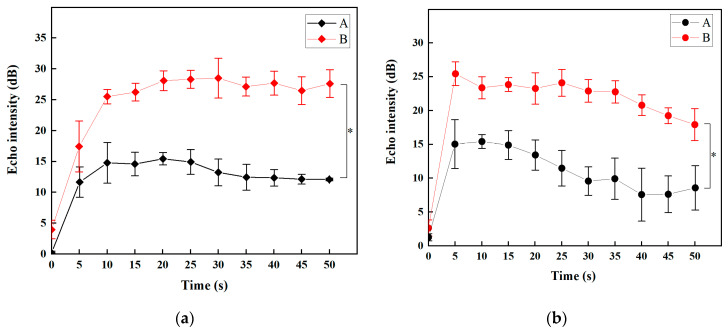
(**a**) Quantitative analysis of the mean echo intensity in the drawn ROIs in SD rat livers after injection. (**b**) Quantitative analysis of the mean echo intensity in the drawn ROIs in SD rat kidneys after injection. The echo intensity in ROI, the rectangle with the area of about 1 mm^2^, accepted quantitative analysis by the data analysis software QLAB10. Group A: tail intravenous injection of SV was set as a control. Group B: tail intravenous injection of SV@rGO-EA. SV concentration in each group was 5 mg/mL. The rGO-EA concentration in SV@rGO-EA MBs was 0.10 mg/mL. The MI value was 0.08. *: *p* < 0.05 compared with each group (*n* = 6).

A quantitative analysis of the mean echo intensity of livers and kidneys in the drawn ROIs in Figure 10 was carried out. The contrast enhancement performance of the MBs in the livers of rats was evaluated by analyzing the acoustic signal intensity within the ROIs located in the liver parenchyma with uniform vascular distribution. Figure 10a reveals that after the injection at 10 s, the echo intensity values of the livers with SV and SV@rGO-EA MBs were 14.8 ± 3.3 dB and 25.5 ± 1.2 dB, respectively. The echo intensity of SV@rGO-EA exhibited a zooming increase in 10 s and stabilized over 40 s, demonstrating almost twice the echo intensity value over SV. The ROIs within the renal cortex of rats were selected to analyze the contrast-enhancing ability in the kidneys. Regarding the echo intensity values of SD rat kidneys in Figure 10b, SV and SV@rGO-EA MBs were 15.0 ± 3.6 dB and 25.4 ± 1.7 dB after the early injection (at 5 s). To sum up, the echo intensity of SV@rGO-EA was significantly stronger than SV in livers and kidneys.

To sum up, rGO-EA nanosheets with a monolayer gauzy structure (Figure 4b) could improve the contrast-enhancing effect of SV@rGO-EA in the liver and kidney of SD rats (Figure 9 and Figure 10), which corresponded with the results from in vitro ultrasound imaging (Figure 8). SV@rGO-EA demonstrated a superior enhancement of US imaging both in vitro and in vivo, making it a much more effective agent for CEUS compared to SV MBs.

## 3. Materials and Methods

### 3.1. Materials

Graphene oxide (GO) was purchased from Sailing Commercial Co., Ltd. (Tianjin, China). Ellagic acid (CAS: 476-66-4) was purchased from Shanghai Macklin Biochemical Technology Co., Ltd. (Shanghai, China). Tris(hydroxymethyl)aminomethane hydrochloride (CAS: 1185-53-1) was purchased from Shanghai Aladdin Bio-Chem Technology Co., Ltd. (Shanghai, China). RPMI 1640 medium (PM150110A) and Dulbecco’s Modified Eagle’s Medium (DMEM, PM150210) were purchased from Procell Life Science & Technology Co., Ltd. (Wuhan, China). Phosphate-buffered saline (PBS) was purchased from Biosharp^®^ (Hefei, China). SonoVue (sulfur hexafluoride microbubbles for injection) was purchased from Bracco Sine Pharmaceutical Co., Ltd. (Shanghai, China).

### 3.2. Cell Lines and Animals

The murine 4T1 cell line (CL-0007) and NIH3T3 cell line (CL-0171) were kindly provided by Procell Life Science & Technology Co., Ltd. (China). The 4T1 cell line was cultured in an RPMI 1640 medium with 10% fetal bovine serum and 100 IU mL^−1^ penicillin/streptomycin. The NIH3T3 cell line was cultured in DMEM medium with 10% fetal bovine serum and 100 IU mL^−1^ penicillin/streptomycin. Female SD rats (6–8 weeks old) with body weights of 150 g were obtained from the Laboratory Animal Center of Zhengzhou University (China).

### 3.3. Synthesis of rGO-EA

For the green synthesis of rGO-EA, 400 mg of EA powder and 100 mg of GO were added to a beaker containing 100 mL distilled water. An appropriate dosage of Tris was mixed into the solution for even distribution, and the pH was adjusted to 8.5. After ultrasonication for 2 h, the well-dispersed solution was stirred in the condition of 70 °C for 24 h. The final product was diluted and washed with deionized water three times to ensure the complete removal of residual EA and Tris. It was freeze-dried for further use.

### 3.4. Characterization of rGO-EA

To determine the characteristics of GO and rGO-EA, a Fourier-transform infrared (FTIR) spectrophotometer (SHIMADZU, IRTracer-100, Tokyo, Japan), ultraviolet–visible (UV–vis) spectrophotometer (SHANGHAIYOUKE, T2602, Shanghai, China), X-ray diffraction (XRD) instrument (Rigaku, D/MAX-gA, Tokyo, Japan), X-ray photoelectron spectrum (XPS) instrument (FEI, ESCALAB 250Xi, Hillsboro, OR, USA) and a thermal gravimetric analysis (TGA) measurement (NETZSCH, STA 449, Selb, Germany) were utilized. Furthermore, a transmission electron microscopy (TEM) microscope (FEI, Tecnai G2 F20 S-TWIN, Hillsboro, OR, USA) was used to confirm the morphology. The zeta potential of the GO and rGO-EA samples was determined five times at 25 °C using a potentiometric analysis instrument (Malvern, Zetasizer Nano ZS90, Worcestershire, UK). 

### 3.5. Synthesis of SV@rGO-EA

Briefly, 5 mL sterile normal saline was added to the SonoVue vial using a syringe, then shaken vigorously for 20 s to mix the contents to obtain SV MBs (5 mg/mL). A SonoVue vial was filled with white lipid powder and sulfur hexafluoride gas. Sterile normal saline was shaken well in the vial to obtain a white milky homogeneous liquid, which contained sufficient SV MBs. SV@rGO-EA MBs were produced after the mixture of rGO-EA saline solution and the homogeneous liquid with SV MBs, which showed a light gray hue. To be specific, 2mg of rGO-EA was added to 5 mL saline and sonicated for 1 h to achieve uniform dispersion. The rGO-EA solution (0.40 mg/mL) was mixed with an equal volume of SV (5mg/mL) to create SV@rGO-EA MBs, with an equivalent concentration of rGO-EA at 0.20 mg/mL. Similarly, vials containing self-assembly SV@rGO-EA MBs were dispersed in saline solution, resulting in equivalent concentrations of rGO-EA at 0.05 and 0.10 mg/mL.

### 3.6. Characterization of SV@rGO-EA

The zeta potential of SV and SV@rGO-EA MBs solutions was analyzed on a Zetasizer Nano ZS90 analyzer (Malvern, UK). The measurement was repeated at least five times, and average values of zeta potential were adopted. A TEM microscope (Thermo Fisher, Talos L120CG2, Brno, Czech Republic) was also employed to observe the structural and morphological characterizations of SV and SV@rGO-EA MBs.

### 3.7. Cell Viability Assay

The growth-inhibiting ability of rGO-EA was preliminarily evaluated using the Cell Counting Kit-8 (CCK-8) assay. The 4T1 and NIH3T3 cells were respectively seeded in 96-well assay plates for 24 h under conditions of 37 °C in a humidified incubator with 5% CO_2_. Subsequently, wells were treated with 200 μL of culture medium containing different concentrations of rGO-EA for an additional 24 h under the same environment. The control wells were simply filled with culture medium and cells. Following three washes of the culture with 200 μL of PBS solution, each well was supplemented with 10 μL of CCK-8 assay and 100 μL of culture medium under identical conditions for 2 h. Cell viability was assessed using a microplate reader (BioTek, Synergy H1, Santa Clara, CA, USA) by measuring absorbance at 450 nm and calculated as a percentage relative to the control (cells grown without the samples were considered to have 100% viability).

### 3.8. Hemolysis Assay

A hemolysis assay was performed to further evaluate the toxicity of the final products. A solution of SV@rGO-EA was prepared with sterile normal saline and kept at 37 °C for 30 min. An experimental group with a 4% RBC suspension (0.5 mL) was added to the SV@rGO-EA suspensions with a concentration gradient (0.5 mL) at 37 °C for 4h. Deionized water or sterile normal saline (0.5 mL) was added to 0.5 mL of 4% RBC suspension as a positive and negative control, respectively. The mixed suspensions were centrifuged at 3000 rpm for 5 min to collect the supernatant. The absorbance values of the supernatants were measured at 545 nm with a microplate reader (BioTek, Synergy H1, USA) to detect the released hemoglobin from the RBCs.

### 3.9. In Vitro Ultrasound Imaging

The plastic pipettes were separated into three groups, filled with each concentration of SV@rGO-EA MBs solution and securely sealed by clamps. The concentrations of SV@rGO-EA solutions were 0.05, 0.10 and 0.20 mg/mL, respectively. The pipette filled with SV MBs served as a control. A large beaker, filled with a thick sound-absorbing sponge at the bottom, was filled with degassed distilled water. The pipettes containing samples were then immersed into the beaker at 25 °C. The ultrasound transducer was maintained parallel to the long axis of the pipettes, which were submerged in water. All the samples underwent scanning using the Hivision Ascendus ultrasound diagnostic system (HITACHI, Tokyo, Japan) in both B-mode and CEUS mode. The MI values were set at 0.10, 0.20, 0.40, 0.60, 0.80 and 1.00, respectively. The focus was adjusted within the range of depths from 1.2 cm to 2.0 cm, while maintaining an imaging target depth of 4 cm. The real-time observation of ultrasound contrast-enhanced imaging was conducted, and the videos were stored.

### 3.10. In Vivo Ultrasound Imaging

SD rats were randomly divided into two groups with four rats in each. After the general anesthesia via an intraperitoneal injection of 10 mg/mL pentobarbital sodium (40 mL/kg), which could be sustained for 2 h, the rats were placed on their backs on heated blankets with their limbs fastened appropriately. The white fur on the chest and belly was depilated.

The in vivo imaging experiments were conducted using a PHILIPS EPIQ 7 ultrasound diagnostic system (PHILIPS, Tokyo, Japan) equipped with a L12-5 linear array transducer (5–12 MHz). In response to the results of ultrasound imaging in vitro, a MI of 0.08 was selected for the contrast-enhanced imaging of MBs in SD rat livers and kidneys. Prior to contrast agent injection, CEUS and B-mode imaging were performed on rats’ livers and kidneys. Then, the control was administered with 200 μL of intravenously injected SV, and the other group received an intravenous injection with 200 μL SV@rGO-EA MBs (with an rGO-EA concentration equivalent to 0.1 mg/mL). At the conclusion of the experiments, euthanasia was performed on the SD rats.

SV with a delicate lipid shell will be significantly disrupted at high MIs. Fortunately, ultrasonic irradiation with a MI of 0.08 can generate an ideal US echo intensity for a considerable duration. To elucidate the alterations in echo intensity, the livers and kidneys were imaged within 50 s following intravenous injection. The echo intensities of the livers and kidneys in rats imaged with each UCA (SV@rGO-EA and SV) were quantitatively analyzed using QLAB10, a specialized quantitative analytical software, in conjunction with the diagnostic color Doppler ultrasound system. The intensity values within specific regions of interest (ROIs) were also subjected to further analysis.

### 3.11. Statistical Analysis

All data were analyzed using IBM SPSS Statistics 22 software. Measurement data were presented as mean ± standard deviation. Student’s *t* test was used for comparisons between two groups. Data among three or more groups were analyzed according to one-way ANOVA followed by a Bonferroni post hoc test. The significance level was considered *p* < 0.05.

## 4. Conclusions

In this work, GO was successfully reduced by ellagic acid, with EA loaded on the surface of rGO. The convenient one-step green reduction of graphene oxide and the adsorption of EA were clarified by various characterization techniques, including UV–vis, FTIR, Raman, XRD, TGA, XPS, TEM and zeta potential analysis. Additionally, the absorption of rGO-EA onto the spherical surface of SV MBs was successfully achieved. The biosafety of the product was confirmed through a cell viability assay and hemolysis assay. The results produced a good biomedical material, enhancing echo intensity and MI tolerance and surpassing the performance of SV MBs both in vitro and in vivo. The application of graphene derivatives in UCAs has been preliminarily investigated, with various graphene derivatives being considered as promising materials for extensive research and development in the medical ultrasound field.

## Data Availability

Data available from Q.C. on personal request.
